# Supplementary material for: Decision-related feedback in visual cortex lacks spatial selectivity
Source: Nat Commun. 2021 Jul 22;12:4473. doi: 10.1038/s41467-021-24629-0 (PMC8298450; doi:10.1038/s41467-021-24629-0)
Supplement: Supplementary file 1 — Supplementary Information [file 41467_2021_24629_MOESM1_ESM.pdf]

Supplementary Information

Decision-related feedback in visual cortex lacks spatial selectivity

Katrina R. Quinn[1], Lenka Seillier[1], Daniel A. Butts[2], Hendrikje Nienborg[3*]

1) University of Tübingen, Germany
2) Department of Biology and Program in Neuroscience and Cognitive Science, University of Maryland, College Park, MD, USA
3) Laboratory of Sensorimotor Research, National Eye Institute, National Institutes of Health, Bethesda, MD, USA

*hendrikje.nienborg@nih.gov

Supplementary Note 1: Psychophysical kernels for bilateral no-signal stimuli

To further confirm that the animals relied correctly on the relevant rather than the irrelevant stimulus we also restricted the analysis of the psychophysical kernels to those trials for which both the relevant and irrelevant stimulus had no net disparity signal (no-signal stimulus, Supplementary Figure 1). Note that because of the reduced number of trials these kernels are noisier but the structure mirrors that of the kernels in Fig. 1.

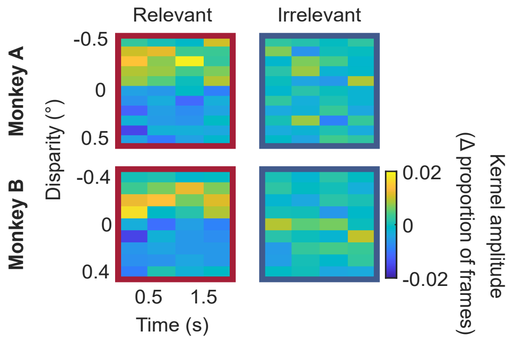

Supplementary Figure 1 | Psychophysical kernels for bilateral no-signal stimuli. For monkey A the kernels were computed for a total of n=1419 trials in 41 sessions, and for monkey B for a total of n=1328 trials in 26 sessions.

Supplementary note 2: GLM analysis of behavior

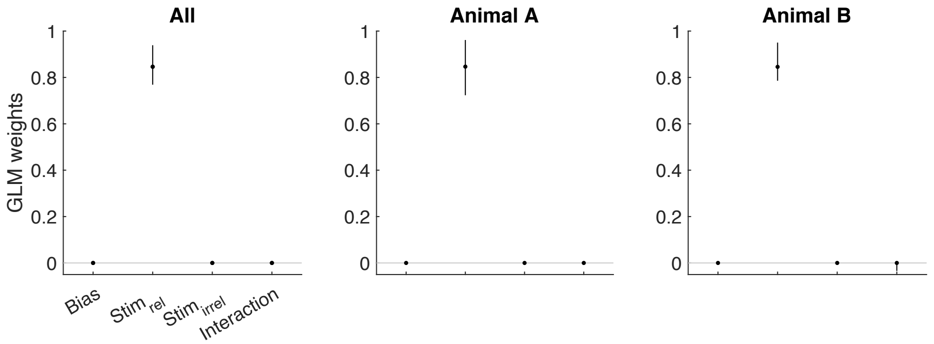

Supplementary Figure 2 | GLM analysis of behavior. GLM weights were obtained for the animals' bias, the relevant stimulus ($Stim_{rel}$), the irrelevant stimulus ($Stim_{irrel}$) and the interaction between the relevant and irrelevant stimulus ($Stim_{rel}*Stim_{irrel}$). The median weights are plotted across all sessions (left panel, n=67 sessions), animal A (middle panel, n=41 sessions) and animal B (right panel, n=26 sessions), respectively. Errorbars are 95% confidence intervals around the median obtained by resampling.

Supplementary Figure 2 shows that hat the median weights for the irrelevant stimulus and the interaction are 0, further supporting the minimal influence of the irrelevant stimulus on the animals' choices.

Supplementary note 3: Choice-correlations for "frozen noise" stimuli

To quantify choice-correlations we corrected stimuli for the random fluctuations of the white-noise stimuli used to perform psychophysical reverse correlation. To verify that this correction was adequate we computed choice-correlations for repeated presentations of the same random sequence of disparity values ("frozen noise") in a subset of sessions. We note that to allow for the psychophysical reverse correlation analysis to verify the spatial selectivity of the behavior, we needed a subset of random noise stimuli even for those sessions in which we included frozen noise stimuli. We therefore fixed the random seeds on half of the trials and randomized them on the other half. But for this reason the number of trials for which choice-correlations were computed was reduced to 50%, and made our estimates of the choice-correlations noisier. Nonetheless, the results of the analyses for the frozen noise stimuli were overall similar to our findings for the corrected choice-correlations (Supplementary Figure 3), and the values of the choice-correlations corrected for stimulus-induced variations were highly correlated with those obtained for frozen noise (V2: $r_{relevant}$=0.46, $p=10^{-13}$, n=239; $r_{irrelevant}$=0.29, $p=10^{-4}$, n=209; V3: $r_{relevant}$=0.71, $p=10^{-20}$, n=143; $r_{irrelevant}$=0.52, $p=10^{-10}$, n=139, Spearman's rank correlations). While the correlation between the choice-correlations for the neurons representing the relevant and irrelevant stimulus, respectively, was reduced to a trend in this subset of the data in V2 (n=209, r=0.10, p=0.14, Spearman's rank correlation), it remained highly significant across the V3/V3a population (r=0.28, p=0.001), similar to the corrected values obtained for the entire population

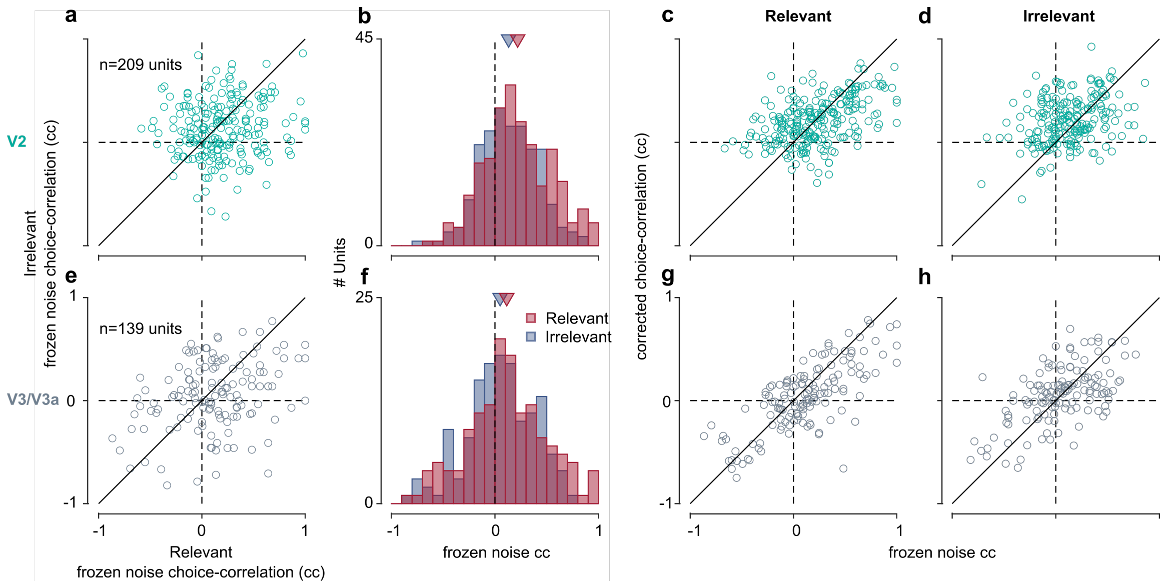

Supplementary Figure 3 | Choice-correlations are similar for identical stimuli ("frozen noise"). a) Choice-correlations (cc) were calculated for trials with a fixed sequence of disparity frames ("frozen noise") and compared for the relevant (abscissa) or irrelevant (ordinate) stimulus. While the correlation was weak for V2 (n=209 units, r=0.10, p=0.14, two-sided Spearman's rank correlation throughout), it was highly significant across the V3/V3a population (**e**, r=0.28, p=0.001), similar to the corrected values obtained for the entire population. **b**) Choice-correlations for frozen noise were significantly positive for both the relevant (mean=0.19, $p=10^{-13}$) and irrelevant stimulus (mean=0.13, $p=10^{-9}$, both two-sided Wilcoxon sign-rank test, uncorrected for multiple comparisons) and comparable, although their medians differed significantly from one another (two-sided Wilcoxon rank sum, p=0.03). **c**) Choice-correlations calculated for frozen noise were similar to those obtained for the white noise corrected for variations in the stimulus for the relevant stimulus (r=0.46, $p=10^{-13}$, n=239 units) and (**d**) irrelevant stimulus (r=0.29, $p=10^{-4}$, n=209 units). **e-h**) Same as **a-d** but for V3/V3a (n=139; relevant: mean=0.12, p=0.001; irrelevant: mean=0.05, p=0.04; difference in distributions, p=0.13; correlation of choice-correlations for frozen stimuli with those corrected for stimulus variations for the relevant stimulus: r=0.71, $p=10^{-20}$, n=143 units and irrelevant stimulus: r=0.52, $p=10^{-10}$, n=139 units).

Supplementary Note 4: Control for eye-movement effects on choice-correlations

Our task and stimulus were designed to minimize potential effects of eye movements that might induce co-variability between choices and neural activity unrelated to the animals' decision process. First, the position of the choice icons was randomized from trial to trial. This ensured that during the stimulus presentation and decision formation period the mapping of the choice to a motor command was unknown to the animal. Second, the disparity noise was designed such as to avoid systematic effects of the animals' vergence eye movements on firing rate. In the control analyses below we verify that these measures were successful in minimizing any effects of eye-movements on the observed choice-correlations.

Effects of mean eye position

Given the randomization of the position of the choice targets an association between the animals' eye-position during the stimulus presentation and choice seemed unlikely. Indeed, there was no significant difference in the animals' eye positions preceding a target presented above or below the fixation marker (mean difference in vertical eye position: Δy: -0.001°, p=0.31; animal A: Δy=-0.0005°, p=0.52, animal B Δy=-0.002°, p=0.30; mean difference in horizontal eye position: Δx: 0.0004°, p=0.57; animal A: Δx=0.0005°, p=0.73, animal B Δx=0.0002°, p=0.79, all two-sided sign-rank tests). We also found little difference in eye position preceding a saccade to the upper or lower target (mean difference in vertical eye position: Δy: -0.0007°, p=0.57; animal A: Δy=0.0003°, p=0.74, animal B Δy=-0.002°, p=0.28; mean difference in horizontal eye position: Δx: 0.002°, p=0.25; animal A: Δx=-0.002°, p=0.09, animal B Δx=0.008°, p=0.002, all two-sided sign-rank tests). As a consequence the difference in the animals' mean eye position as a function of choice were small (mean difference in horizontal eye position preceding near compared to far choices: Δx: -0.007°, p=0.82; animal A: Δx=-0.002°, p=0.002, animal B Δx=-0.01°, p=0.001; mean difference in vertical eye position: Δy: -0.02°, p=0.08; animal A: Δy=-0.02°, p=0.81, animal B Δy=-0.013°, p=0.04 all two-sided sign-rank tests). To verify that these differences did not account for the choice-correlations we observed we measured the effects of mean vertical or horizontal eye position on firing rate within each choice and then regressed out the predicted effect across choices. These corrected choice- correlations were very similar to the uncorrected values (mean corrected choice-correlation and correlation with the uncorrected values in V2: correction for horizontal eye position: $cc_{relevant}$=0.11, r=0.97, $p=10^{-20}$, $cc_{irrelevant}$=0.07, r=0.97, $p=10^{-20}$; correction for vertical eye position: $cc_{relevant}$=0.11, r=0.99, $p=10^{-20}$, $cc_{irrelevant}$=0.07, r=0.96, $p=10^{-20}$; in V3: correction for horizontal eye position: $cc_{relevant}$=0.09, r=0.99, $p=10^{-20}$, $cc_{irrelevant}$=0.07, r=0.98, $p=10^{-20}$; correction for vertical eye position: $cc_{relevant}$=0.09, r=0.99, $p=10^{-20}$, $cc_{irrelevant}$=0.07, r=0.96, $p=10^{-20}$) indicating that differences in eye position with choice do not account for the choice-correlations we observed.

Effects of microsaccades

Microsaccades have been shown to weakly modulate the activity of visual neurons (e.g. [1,2]). We therefore explored any systematic relationship between the frequency, amplitude and direction of microsaccades and choice-correlations. Microsaccades were labeled using a recently developed algorithm [3]. While there were no differences in amplitude or direction with choice (p=0.21, p=0.84, respectively), microsaccades occurred with slightly higher frequency on trials preceding far choices (1.2 vs 1.4Hz, $p=10^{-8}$, all two-sided sign-rank tests). If this difference contributed to the observed choice-correlations it would predict higher choice-correlations on sessions for which the choice-dependent difference in microsaccades was more pronounced. In contrast to this prediction, we observed a negative correlation between choice-correlation and |difference in microsaccade

frequency with choice| for units in V2 but not V3, V3a (V2: $r=-0.20$, $p=10^{-7}$, $r=-0.12$, $p=0.001$, correlation with choice-correlation for the cued and uncued stimulus, respectively; respective values for V3/V3a: $r=0.07$, $p=0.14$, $r=-0.05$, $p=0.28$; all Spearman's rank correlation). Choice-correlations therefore tended to be higher for sessions in which microsaccades were balanced across choices. This shows that rather than contributing to choice-correlations in V2, differences in the microsaccade frequency with choice may have obscured stronger correlations with choice on some sessions.

Effects of vergence

We note that contrary to a stimulus without disparity noise for which small con- or divergence eye movements could result in systematically different effects on firing rate for near and far-preferring neurons cf. [4], the disparity noise in our stimulus was drawn from a wide distribution of values and designed to avoid such systematic effects. The disparity noise had a distribution of values that typically well exceeded the peak of the disparity tuning curve of the recorded units. Differences in the animals' vergence with choice therefore could have inconsistent effects on the neuronal responses to the noise stimulus depending on their tuning curves. In control analyses we examined whether any choice-related differences in vergence may have contributed to the choice-correlations we observed, in particular to the choice-correlations for the irrelevant stimulus. Similar to previous studies, both animals had a tendency to converge slightly more on trials for which they made near reports (mean vergence difference for near compared to far choices across sessions: $v=-0.012°$, $p=10^{-5}$; animal A: $\Delta v=-0.016°$, $p=10^{-4}$, animal B $\Delta v=-0.009°$, $p=0.029$, all two-sided sign-rank tests). The resulting difference in vergence means that when the animal chose near the stimulus disparities had a tendency to be slightly shifted towards far values. The direction of this shift was therefore the opposite direction to what one would expect could result in the correlations with choice we observed. To examine any potential effect of the small difference in vergence across choices without making assumptions about the underlying tuning curves we directly measured the change in firing rate with vergence within choice. We then regressed out the vergence-dependent change in firing from the spike count for each trial and unit and re-computed choice- correlations for these corrected values. This correction resulted in very similar but on average slightly increased values for choice-correlations compared to the uncorrected values (corrected mean choice-correlation and correlation with the uncorrected values in V2: $cc_{relevant}=0.115$, $r=0.96$, $p=10^{-20}$, $cc_{irrelevant=}$ 0.088, $r=0.95$, $p=10^{-20}$; in V3: $cc_{relevant}=0.075$, $r=0.94$, $p=10^{-20}$, $cc_{irrelevant=}$ 0.072, $r=0.91$, $p=10^{-20}$), as expected from the direction of the vergence difference. It shows that the choice-correlations we measured are not accounted for by vergence eye movements but rather that due to the animals' vergence we may have slightly underestimated the neuronal correlations with choice.

Supplementary Note 5: additional experimental information.

Here we show the average receptive field positions for each session (Supplementry Figure 4), and example data for recordings in each animal in V2 and V3/V3a (Supplementary Figure 5).

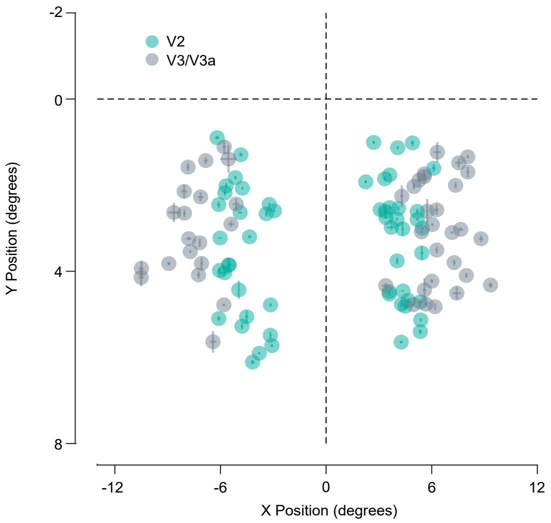

Supplementary Figure 4 | Receptive field centers. Average receptive field positions (in °) are shown for each included session for V2 (green) and V3/V3a (gray) and monkeys A and B. For each penetration, horizontal and vertical receptive field profiles were measured from the responses on each channel using an elongated grating or bar, and fit by Gaussian functions. The average center and width of the receptive fields was computed from the mean and SD of fits which explained >70% of the variance (V2 mean horizontal center=4.48°, width=±1.04°, vertical center=3.38°, width=±0.95°; V3/V3a mean horizontal center=6.83°, width=±1.54°, vertical center=3.05°, width=±1.35°).

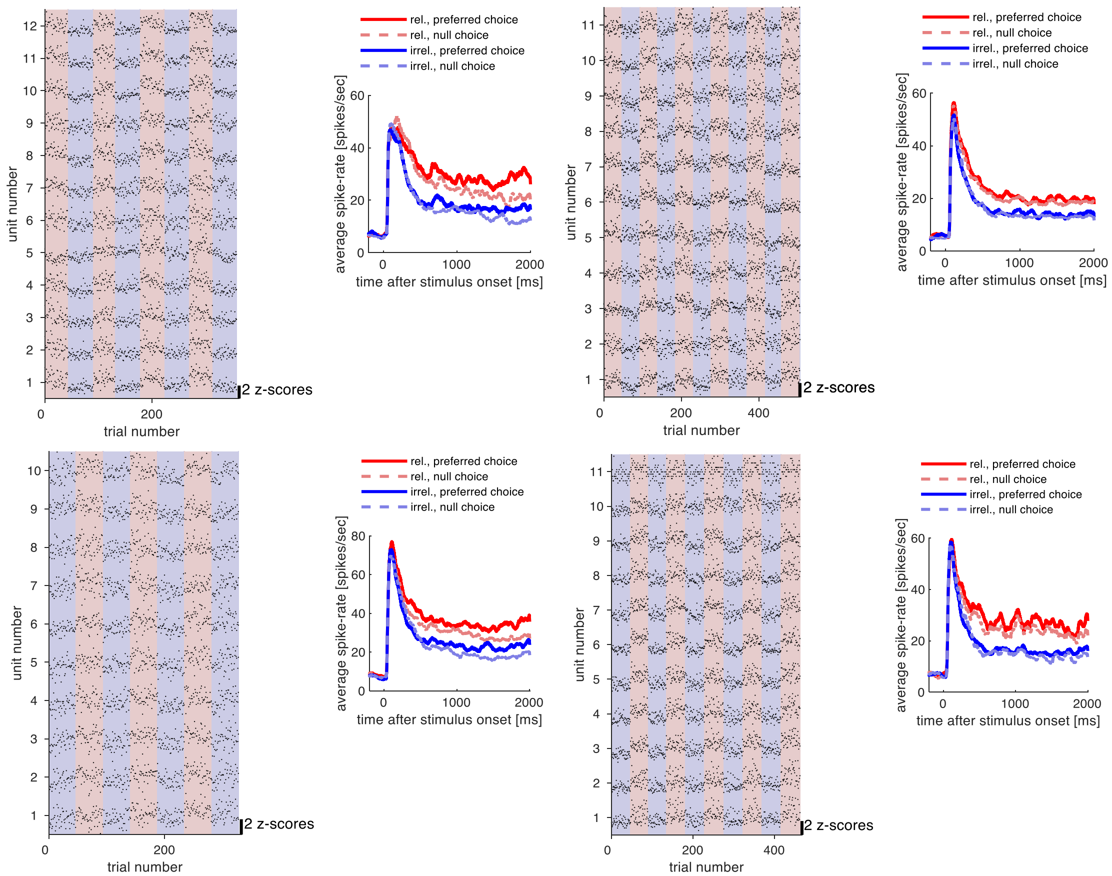

Supplementary Figure 5 | Example recordings. Top row: recordings in V2. Bottom row: recordings in V3. Left column: animal A. Right column animal B. Left panel: For each unit, the spike counts per trial (converted to z-scores) are plotted for each trial. Trials on which the stimulus inside the receptive field was relevant (irrelevant) are shaded in red (blue). Right panel: the average responses across units to a 0% signal stimulus, separated by relevance condition and the animal's choice.

Supplementary References

1. Leopold, D. A. & Logothetis, N. K. Microsaccades differentially modulate neural activity in the striate and extrastriate visual cortex. *Exp Brain Res* **123**, 341-345 (1998).
2. McFarland, J. M., Bondy, A. G., Saunders, R. C., Cumming, B. G. & Butts, D. A. Saccadic modulation of stimulus processing in primary visual cortex. *Nat Commun* **6**, 8110 (2015).
3. Bellet, M. E., Bellet, J., Nienborg, H., Hafed, Z. M. & Berens, P. Human-level saccade detection performance using deep neural networks. *J Neurophysiol* **121**, 646-661 (2019).
4. Clery, S., Cumming, B. G. & Nienborg, H. Decision-related activity in macaque V2 for fine disparity discrimination is not compatible with optimal linear read-out. *J Neurosci* **37**, 715-725 (2017).
