# Supplementary material for: Decision-related feedback in visual cortex lacks spatial selectivity
Source: Nat Commun. 2021 Jul 22;12:4473. doi: 10.1038/s41467-021-24629-0 (PMC8298450; doi:10.1038/s41467-021-24629-0)
Supplement: Supplementary file 3 — Reporting Summary [file 41467_2021_24629_MOESM3_ESM.pdf]

nature portfolio

Corresponding author(s): Hendrikje Nienborg

Last updated by author(s): Jun 9, 2021

# Reporting Summary

Nature Portfolio wishes to improve the reproducibility of the work that we publish. This form provides structure for consistency and transparency in reporting. For further information on Nature Portfolio policies, see our Editorial Policies and the Editorial Policy Checklist.

## Statistics

For all statistical analyses, confirm that the following items are present in the figure legend, table legend, main text, or Methods section.

| n/a | Confirmed | |
|---|---|---|
| ☐ | ☒ | The exact sample size (*n*) for each experimental group/condition, given as a discrete number and unit of measurement |
| ☐ | ☒ | A statement on whether measurements were taken from distinct samples or whether the same sample was measured repeatedly |
| ☐ | ☒ | The statistical test(s) used AND whether they are one- or two-sided<br>*Only common tests should be described solely by name; describe more complex techniques in the Methods section.* |
| ☐ | ☒ | A description of all covariates tested |
| ☐ | ☒ | A description of any assumptions or corrections, such as tests of normality and adjustment for multiple comparisons |
| ☐ | ☒ | A full description of the statistical parameters including central tendency (e.g. means) or other basic estimates (e.g. regression coefficient) AND variation (e.g. standard deviation) or associated estimates of uncertainty (e.g. confidence intervals) |
| ☐ | ☒ | For null hypothesis testing, the test statistic (e.g. *F*, *t*, *r*) with confidence intervals, effect sizes, degrees of freedom and *P* value noted<br>*Give P values as exact values whenever suitable.* |
| ☒ | ☐ | For Bayesian analysis, information on the choice of priors and Markov chain Monte Carlo settings |
| ☒ | ☐ | For hierarchical and complex designs, identification of the appropriate level for tests and full reporting of outcomes |
| ☐ | ☒ | Estimates of effect sizes (e.g. Cohen's *d*, Pearson's *r*), indicating how they were calculated |

*Our web collection on statistics for biologists contains articles on many of the points above.*

## Software and code

Policy information about availability of computer code

| | |
|---|---|
| Data collection | Electrophysiological data was collected using Trellis (Ripple Neuro). Behavioral control and stimulus generation was done using custom Matlab code based on Eastman & Huk (2012) and Psychtoolbox (PTB3). |
| Data analysis | Spike sorting was done using Plexon Offline Sorter (v3.3.5). Analysis using Matlab (Mathworks, R2019b) and custom python code is available at: https://github.com/NienborgLab/Quinn_et_al_2021. |

For manuscripts utilizing custom algorithms or software that are central to the research but not yet described in published literature, software must be made available to editors and reviewers. We strongly encourage code deposition in a community repository (e.g. GitHub). See the Nature Portfolio guidelines for submitting code & software for further information.

## Data

Policy information about availability of data

All manuscripts must include a data availability statement. This statement should provide the following information, where applicable:

- Accession codes, unique identifiers, or web links for publicly available datasets
- A description of any restrictions on data availability
- For clinical datasets or third party data, please ensure that the statement adheres to our policy

Source data to replicate the findings of the study are available at https://github.com/NienborgLab/Quinn_et_al_2021.

# Field-specific reporting

Please select the one below that is the best fit for your research. If you are not sure, read the appropriate sections before making your selection.

- [x] Life sciences
- [ ] Behavioural & social sciences
- [ ] Ecological, evolutionary & environmental sciences

For a reference copy of the document with all sections, see nature.com/documents/nr-reporting-summary-flat.pdf

# Life sciences study design

All studies must disclose on these points even when the disclosure is negative.

| | |
|---|---|
| Sample size | Sample size for the main dataset was 703 units in V2 and 486 units for V3/V3a from two rhesus macaque subjects. Two rhesus macaque monkeys is typical for comparable studies (e.g. Nienborg & Cumming, 2009, Nature), and the number of units exceed similar prior studies in awake monkeys. |
| Data exclusions | Only successfully completed trials were included for further analysis. For each unit periods of pronounced non-stationarity were removed. To do so we computed a running 20-trial average spike-count, calculated separately for each attention condition. Periods for which this running average decreased below 20% its peak were removed, and the longest continuous segment of included trials was used for further analysis. This resulted in the removal of 8 (0.6%) of the V2 units and 24 (2%) of the V3/V3a units because the remaining data after removing periods of non-stationarity did not meet the minimum number of trials to compute choice-correlations (at least 5 trials with near and far choices, respectively for the 0% signal stimulus). Additionally, only units for which the mean response to the 0% signal stimulus exceeded 4 spikes/sec, and for which the d-prime to discriminate the stimuli with the highest signal strength was >0.5 were included. Additionally, the following behavioral criteria had to be met for each unit: the animal's performance had to exceed 70% for the highest signal stimuli and the bias for 0% signal trials had to be below 75%. For the V2 dataset, out of 1301 units 152 (12%), 266 (20%) and 172 (13%) were excluded because the criteria for minimum firing rate, d-prime or behavior, respectively, were not met. For V3/V3a, out of 1035 units, 123 (12%), 298 (29%) and 104 (10%) were removed for these reasons, respectively. In addition to the inclusion criteria applied to the main dataset, the population vector analysis excluded units that had a majority of their response (>50%) explained by the drift term in its encoding model. Following this additional screening, only recordings with five or more valid units per area were included in the resulting measurements of population vectors. For the included units the GLM accounted for an average of 52% (V2) and 50% (V3/V3a) of the variance (five-fold cross-validation). |
| Replication | The main results were reliably replicated in two monkeys, which is standard in non-human primate models. |
| Randomization | All comparisons were made within subject across task conditions, i.e. each subject was their own control. |
| Blinding | The investigators were not blinded but initial experimental conditions were randomized and stimuli presented randomly as determined by a computer. |

# Reporting for specific materials, systems and methods

We require information from authors about some types of materials, experimental systems and methods used in many studies. Here, indicate whether each material, system or method listed is relevant to your study. If you are not sure if a list item applies to your research, read the appropriate section before selecting a response.

## Materials & experimental systems

| n/a | Involved in the study | |
|---|---|---|
| ☒ | ☐ | Antibodies |
| ☒ | ☐ | Eukaryotic cell lines |
| ☒ | ☐ | Palaeontology and archaeology |
| ☐ | ☒ | Animals and other organisms |
| ☒ | ☐ | Human research participants |
| ☒ | ☐ | Clinical data |
| ☒ | ☐ | Dual use research of concern |

## Methods

| n/a | Involved in the study | |
|---|---|---|
| ☒ | ☐ | ChIP-seq |
| ☒ | ☐ | Flow cytometry |
| ☒ | ☐ | MRI-based neuroimaging |

## Animals and other organisms

Policy information about studies involving animals; ARRIVE guidelines recommended for reporting animal research

| | |
|---|---|
| Laboratory animals | Two pair-housed male adult rhesus monkeys (Macaca mulatta), both aged 13 years were used. |
| Wild animals | No wild animals were used in this study. |
| Field-collected samples | No field collected samples were used in the study. |
| Ethics oversight | All experimental protocols were approved by the relevant local authorities (Regierungspraesidium Tuebingen, Germany). |

Note that full information on the approval of the study protocol must also be provided in the manuscript.
